# Young Child-Rearing Latina Cancer Survivors Living in the US-Mexico Border Region: A Qualitative Study

**DOI:** 10.4236/jct.2021.124018

**Published:** 2021-04-22

**Authors:** Clara L. Reyes, Rebecca L. Palacios, Karoline Sondgeroth, Ernesto A. Moralez

**Affiliations:** 1 Department of Public Health Sciences, New Mexico State University, Las Cruces, NM, USA; 2 Department of Public Health, St. Lawrence University, Canton, NY, USA

**Keywords:** Cancer Survivorship, Latinas, Coping, Family, US-Mexico Border

## Abstract

**Background:**

Despite increasing cancer incidence among young Latinas (<50 yrs.) in the US, little is known about how young, child-rearing Latinas cope with cancer in the US-Mexico border region.

**Objective:**

The purpose of this study was to explore how young, child-rearing Latinas described their challenges, strengths, and social support sources for coping with cancer in the US-Mexico border region.

**Methods:**

Nine Latinas that had been diagnosed with cancer, had at least one child 5 to 13 years old, and lived in one of two targeted border counties participated in audio-recorded, semi-structured focus groups (n = 6) or interviews (n = 3) in their preferred language (*i.e.*, English or Spanish). Interview recordings were transcribed and inductively coded using methods based on grounded theory.

**Results:**

Three major themes emerged. First, in reporting their physical and emotional struggles with cancer as the most difficult time of their lives, participants described feeling alone as they navigated treatment side effects and continued fear of cancer. Second, they explained figuring out how to live day-by-day, reporting the negative impact of cancer on their families and on their ability to maintain their roles as mothers. Third, they highlighted factors that gave them the strength to fight and carry on, emphasizing their children and their inner strength.

**Conclusions:**

Even with a supportive family, young Latina mothers felt alone as they navigated cancer (*i.e.*, treatment, fear, and impact on their families) and as they worked to garner the strength to overcome the stress of cancer. Interventions for young Latina survivors should be designed to address their needs, build on their fighting spirit, incorporate the family, and connect them with other survivors for personalized support. Further research is warranted to better understand cancer survivorship among child-rearing Latina mothers experiencing a cancer diagnosis in under-resourced communities like the US-Mexico border region.

## 1. Introduction

Latinas are more likely to be diagnosed with cancer at younger ages (<50 yrs.) and in later stages compared to their non-Latina White (NLW) counterparts [1]. Research shows that younger survivors in the US experience greater financial hardship associated with a cancer diagnosis compared to older survivors, as they have less time to be employed and accrue financial assets [2]. Financial hardships (e.g., poverty and uninsurance) and limited access to cancer treatment are also more pervasive among US-Mexico border residents compared to non-border residents [3]. Thus, young Latina survivors experiencing low socioeconomic status (SES) and residing in the border region may be especially vulnerable to low quality of life and greater cancer mortality following a cancer diagnosis [4].

Despite increased cancer incidence in young Latinas (<50 yrs.) in the US [5], little is known about the challenges they face in managing a cancer diagnosis. Current knowledge of their cancer experiences is inferred from studies conducted with more mature Latinas (*i.e.*, mean age > 50 yrs.) [6] [7] [8] [9] [10]. Studies with older Latinas show they are more vulnerable to experiencing negative cancer outcomes, including low quality of life and high rates of distress [9] [10] and depression than NLW survivors [6]. Challenges impacting these Latinas’ ability to cope with the stress of cancer included economic issues (e.g., family welfare), systemic barriers to quality care (e.g., insurance, undocumented status), persistent medical concerns (e.g., fear of cancer progressing), cultural factors (e.g., fatalism, familism or importance of the family), and personal concerns (e.g., body image, sexual health) [11]-[18].

Whether young Latina survivors experience similar cancer-related challenges and negative outcomes as do more mature Latina survivors remains an empirical question. The only available evidence of survivorship in young mothers comes from research with NLW breast cancer patients (<45 yrs.) of high SES, which suggests they experience lower physical and psychological well-being and greater illness intrusiveness when compared to more mature NLW survivors (>45 yrs.) [19]. Furthermore, young mothers reported greater fears of breast cancer recurrence and interference with partner relationships compared to women without children [19]. Overall, motherhood appeared problematic for the well-being of breast cancer survivors, especially among those younger, closer to the time of diagnosis, experiencing high parenting stress, and having adolescent children [19].

If young Latina cancer survivors in the US-Mexico border region have similar negative cancer experiences as more mature Latina survivors and young NLW survivors, they may be particularly predisposed to poor cancer outcomes. To date, no studies have focused on the experiences or needs of young (<50 yrs.), child-rearing Latina cancer survivors. The purpose of this study was to explore young (<50 yrs.) Latina mothers’ challenges, strengths, and sources of social support in coping with a cancer diagnosis in the border region.

## 2. Methods

### 2.1. Procedure

This qualitative study was approved by the New Mexico State University IRB (#15032). Eligibility criteria included being a Latina mother, diagnosed with cancer of any type, parenting a school-aged child (*i.e.*, 5 – 13 yrs.), and living in either Doña Ana County, NM or El Paso County, TX. Recruitment through flyers, media announcements (e.g., Facebook, newspaper), medical institutions, non-profits, and cancer support groups occurred in the target border counties. Participants were invited to join in 90-minute semi-structured interviews in their preferred format (*i.e.*, focus groups or individual interviews) and in their preferred language (*i.e.*, English or Spanish) to share their experiences and needs as a mother living with cancer.

All semi-structured interviews were led by the bilingual Principal Investigator and all study materials were provided in the participants’ preferred language. Participants first provided written consent and then completed a brief survey assessing their demographics and illness characteristics. The moderator explained the purpose of the study and that the discussion would be audio-recorded before administering a 7-question guide assessing coping challenges, strengths, and sources and types of social support following a cancer diagnosis (Table 1). Participants received a $25-gift-card, a meal, and a cancer resource list.

Two focus groups and three individual interviews were conducted between November 2016 and March 2017. Focus groups were held at the City of El Paso Department of Public Health in El Paso, TX (n = 3 participants) and the Southern Area Health Education Center in Las Cruces, NM (n = 3 participants). Individual interviews (n = 3 participants) were held at locations convenient to participants (e.g., community centers, home, work office) in the targeted border counties.

While the funded project proposed recruiting 20 participants, time constraints and difficulties in recruiting young cancer-diagnosed Latina mothers overburdened with other priorities (e.g., work, family, and cancer demands) ultimately dictated the sample size. However, the small focus groups and individual interviews permitted young Latina mothers to engage in greater reflection and dialogue, thereby providing an extensive description of their cancer experiences and needs. The richness and complexity of the data provided significant insight into the cancer experience of this underrepresented population.

### 2.2. Analysis

Audiotapes were transcribed; Spanish transcripts were translated into English and back-translated to confirm accuracy before coding. Three research staff used inductive coding methods adapted from grounded theory (Table 2) to discover Latina mothers’ cancer experiences [20] [21] [22]. Using ATLAS.ti software, data was unitized and then units were grouped by shared characteristics [20] [21]. Through constant comparative analysis, coders ensured units were appropriately categorized and that the resulting categories were unique [20] [21] [22]. Coders then grouped similar categories into domains (*i.e.* broader conceptual categories) and conducted a comparative analysis of categories within and between each domain [21]. Finally, data were reexamined to determine a core construct, which reflected the major social process through which the young Latina mothers were coping with their cancer while concurrently raising their children [21]. Coding to consensus, formal peer debriefing, and maintaining an audit trail for each phase of the analysis protected the trustworthiness of study results [20] [22] [23].

## 3. Results

Two focus groups and three individual interviews were conducted with nine young (mean = 36.7 yrs.; SD 6.9) Latinas diagnosed with breast (56%; n = 5), thyroid (22%; n = 2), or ovarian cancer (22%; n = 2) within the last three years (mean = 1.9 yrs. since diagnosis; SD 1.1). Most were diagnosed before age 40 (89%; n = 8) (mean = 34.8 yrs.; SD 7.1). Only two participants preferred to speak Spanish. Participants’ education levels ranged from high school or less to a bachelor’s degree. Most were married (67%; n = 6) and employed (67%; n = 6), with 56% (n = 5) living in Doña Ana County, NM and 44% (n = 4) living in El Paso County, TX (Table 3). The analysis yielded three major domains, as described below.

### 3.1. Having the Most Difficult Time of My Life

#### 3.1.1. Feeling Like the Treatment Is Worse Than the Disease

Latina mothers reported physical and emotional struggles in dealing with cancer. They discussed how the treatment was worse than the disease and described long-lasting side effects after treatment termination, such as “chemo brain” and anxiety disorders. Although informed of their disease type and treatments, they did not anticipate the severity of the side effects. “I don’t feel like I was really prepared or educated very well for after-effects.” Some were disturbed by their altered self-image following treatment-related hair loss.

#### 3.1.2. Feeling Very Alone

Mothers reported coping with their cancer fear privately to shield their families from their vulnerability. They described fearing cancer recurrence and uncertainty over the long-term outcome. “They always say it can come back, but even harder, stronger.” Mothers highlighted the difficult transition from treatment to post-treatment, feeling cut-off by their medical teams and support networks. They attributed the lessened attention post-treatment to family and friends’ inability to understand the cancer survivor’s experience (e.g., fear of cancer recurrence). Consequently, mothers reported serving as their own best support. “I think just me … really learning how to hold my own hand.” Mothers expressed the need for continued care, support, and positive energy from family, friends, and medical providers throughout their survivorship, not just through the cancer treatment phase.

### 3.2. Figuring out How to Live Day-by-Day

#### 3.2.1. Being Especially Challenging on My Family and Home Life

Mothers reported needing to be strong for their children and families. Their greatest challenge was not being able to be the mother they used to be. “Not being able to … hold on to the one thing I wanted to, that made me feel normal … was really hard.” Mothers were distressed by the burden placed on their children (e.g., worrying about their mothers) and struggled to describe cancer in ways that would not frighten their children. “It’s hard to explain that to a seven-year-old.” Although families helped significantly, mothers remained concerned about a lack of supervision and discipline for their children. Mothers reported needing additional childcare support, education on how to communicate with their children about cancer, and services to enhance their family’s emotional well-being.

#### 3.2.2. Needing Other Things

Mothers identified several unmet needs that made their cancer experience more trying. Mothers’ inability to work during treatment exacerbated financial burdens for her family. Participants with thyroid and ovarian cancer lacked access to local cancer specialists and struggled with long travel distances, healthcare approval processes to access treatment, and a need for additional childcare resources during their travel. Although participants reported that their families tried to help them as much as they could, their families frequently lacked the time and financial resources to meet all their needs given their own household and work demands. Participants also reported mixed support from their providers, with some providers lacking compassion when delivering treatment decisions or dismissing persistent side-effects.

### 3.3. Giving Me the Strength to Fight and Carry on

#### 3.3.1. Relying on Friends, Family, and My Children for Normalcy, Motivation, and Care

Participants described sources of strength that allowed them to move forward despite cancer-related challenges. Friends and family provided instrumental and emotional support. “Something so horrible happened, but … I got slapped with love. I mean I had so much support. So much family.” Mothers often served as caregivers for the participants and provided childcare for the grandchildren. Participants’ partners also served as caregivers as well as confidantes for cancer care decisions. However, participants described their children as their most significant source of support. Children provided a sense of normalcy by including their diagnosed mother in their daily activities and preserving her role as “mom.” “They never treated me as though I was sick. I was still mom.” Children also motivated them to overcome cancer. “The strength I get from my children … this is my reason to … fight and to carry on.” When adults were not available, children served as their mother’s caregivers and learned to care for themselves and their siblings. “My little one, she would … read to me or get my water or … make sure that … I was ok while my husband took a shower or went to the store.”

#### 3.3.2. Getting Support from Other Cancer Patients Going through It

Participants reported how chance encounters with other cancer survivors (e.g., during chemo or cancer survivor events) offered information, connection, understanding, and continued support. “I would talk to the people around me [during chemo] and that’s what helped me realize, ‘Oh, there is such a thing as chemo brain.’” Some mothers described how assisting in cancer organizations and events helped them to meet and learn from others at different stages in their cancer survivorship. Only one participant reported engaging in cancer support groups.

#### 3.3.3. Not Letting Cancer Stop Me

Participants described personal strategies for coping with cancer, including staying mentally strong and positive to overcome the fear of cancer. “If you let your diagnosis … get to your head, you’re going to lose the battle … You have to be mentally stronger.” They also tried to live as “normally” as possible (e.g., working if they could). A few felt it was essential to make light of the cancer, while others turned to God to help them cope.

## 4. Discussion

Young Latina mothers (<50 yrs.) living in the US-Mexico border region identified various challenges to coping with cancer previously identified in the literature. These challenges included barriers to quality care (e.g., traveling long distances) [3] [11] [24], persistent medical concerns (e.g., long-lasting side effects) [11] [12] [18], economic issues (e.g., unable to work during treatment) [11] [12] [18], personal concerns (e.g., self-image) [6] [11] [12] [14] [18], and cultural familial factors (e.g., being slapped with love from family) [11] [12] [15] [17] [18]. Similar to previous studies in NLW breast cancer survivors, Latina mothers in our study expressed needing to be strong for their school-aged children, struggling with not knowing how to communicate with their children about cancer, and experiencing emotional distress when they were unable to be the mother they used to be [25]. Young Latina mothers also identified coping strategies previously identified in the literature, with their determination to beat cancer resembling the fighting spirit [26] and self-reliance [17] documented in older Mexican American and immigrant Latina breast cancer survivors.

Unique findings in the present study relate to the mother-child relationship and perceived isolation post-treatment of young Latina mothers living with diverse cancers in the US-Mexico border region. Participants in our study described a complex mother-child relationship that impacted their cancer coping. Unlike previous findings among NLW survivors, Latina mothers identified their school-aged children as their greatest source of support, motivation, and strength in coping with cancer. Although school-aged children (5 – 13 years old) occasionally needed to serve as caregivers for their mothers, they managed to include her in ordinary parenting roles (e.g., homework), ultimately helping to provide a sense of normalcy and to preserve her role as mom. Therefore, while struggling to fulfill parenting roles caused significant stress, young Latina mothers also emphasized that their children were their greatest source of strength in fighting the stress of cancer. Connecting diagnosed Latina mothers to programs that assist with childcare, support their children’s emotional wellbeing, and provide education on how to communicate about cancer with their children would help improve the quality of survivorship for both mother and child.

Although Latina mothers received significant support from their families during treatment, they described experiencing abandonment and severe isolation following treatment completion. Furthermore, they felt family and friends were unable to understand their continued fear of cancer recurrence, having never experienced cancer themselves. Reduced contact from providers and social networks after treatment also left Latina mothers feeling isolated and fearful in their continued fight against cancer. They reported needing continued care, support, and positive energy from family, friends, and medical providers throughout their survivorship. Findings reinforce the need to evaluate Latina survivors’ perceived isolation and support needs throughout survivorship [18].

In contrast to their friends, family, and providers, other cancer survivors served to alleviate the Latina mothers’ cancer fears and isolation, providing them with needed connection, understanding, and inspiration [12]. However, only one mother reported participating in a cancer support group, with all others describing only chance encounters with other cancer survivors. Physicians, patients, and patients’ families should receive education on the therapeutic value of connecting survivors (e.g., support groups; cancer buddies) to reduce fear and isolation and improve the quality of survivorship among Latinas [27].

### 4.1. Limitations

The small sample size of the study is a limitation that may be attributed to difficulties in recruiting young diagnosed Latina mothers who are overburdened with other priorities including work, financial, and family demands compounded by a cancer diagnosis. Another potential limitation is the diverse cancer diagnoses of the study participants (*i.e.*, breast, thyroid, and ovarian cancer), with most prior studies focusing exclusively on breast cancer survivors. Interestingly, young Latinas with diverse cancer diagnoses revealed both common and unique needs and concerns. Although these results are preliminary, they suggest we need to expand our understanding of the experiences of young Latina survivors, including those with diagnoses unrelated to breast cancer who are missing from the cancer literature.

### 4.2. Implications

The Latino cultural construct of familism emphasizes the importance of family as an emotional support system which may limit help-seeking behavior outside of the family [28]. In Latinas diagnosed with cancer, familism has been found to serve as a source of stress, motivation, and support [12] [13] [16] [18]. Participants in our study further revealed the complexity of familism during and after cancer treatment. During treatment, participants experienced overwhelming instrumental and emotional support from their families, especially their children. However, once treatment was completed, participants reported reduced contact from family. Reduced contact was attributed to the family not understanding that her fight against the cancer was not over. As she was no longer surrounded by her medical team, participants reported needing their family’s support more than ever. Although they felt isolated, most participants reported not participating in cancer support groups, perhaps given Latino’s tendencies to not seek support outside of the family. With previous studies suggesting Latinas are less likely to attend support groups [27], we need additional research on how young Latinas prefer to connect with other cancer survivors so they can benefit from this invaluable resource. As motherhood is intertwined with familism, we learned that parenting young children following a diagnosis simultaneously causes stress and provides strength to fight cancer for Latina mothers. Additional research on how parenting young children impacts survivorship in Latinas is warranted. In summary, interventions to improve the quality of survivorship for young diagnosed Latina mothers need to engage and foster their fighting spirit, incorporate familism, and create connections with other survivors who can provide personalized and continued support throughout their survivorship.

## Figures and Tables

**Table 1.** Focus group and individual interview guide.

1. What, if anything, has been particularly difficult or challenging for you in coping with your cancer?
2. What are things that have helped your recovery or coping the most?
3. What type of family/friend support, if any, was available to you following your cancer diagnosis?
4. Who gave you the most support to help cope with your cancer?
5. With what did your friends/family help you following your diagnosis?
6. What support did you want that you did not get from family/friends?
7. In what ways, if any, did you rely on your school-age/adolescent children for your support during the cancer treatment and early post-treatment period?

**Table 2.** Summary and sequence of data analytic steps [21].

Step 1: Establish units of analysis of transcribed data
Step 2: Peer debrief the units
Step 3: Inductively code units of analysis into non-overlapping subcategories
Step 4: Peer debrief subcategories
Step 5: Refine and group similar subcategories, ensuring they are mutually exclusive
Step 6: Peer review higher-order groups of subcategories (*i.e.* categories)
Step 7: Group similar categories into domains
Step 8: Peer debrief domains
Step 9: Identify a core construct
Step 10: Peer debrief core construct

**Table 3.** Participant characteristics (n = 9).

Characteristic	Total (Percentage)
Language	
English	7 (78%)
Spanish	2 (22%)
County	
Doña Ana, NM	5 (56%)
El Paso, TX	4 (44%)
Age (yrs.) (mean = 36.67)	
<30	1 (11%)
30 – 39	4 (44%)
40 – 49	4 (44%)
Age at diagnosis (yrs.) (mean = 34.78)	
<30	2 (22%)
30 – 39	6 (67%)
40 – 49	1 (11%)
Education	
High school or less	2 (22%)
Some college, no degree	2 (22%)
Associate or Bachelor’s degree	5 (56%)
Marital Status	
Married	6 (67%)
Single or Divorced	3 (33%)
Employed	
Yes	6 (67%)
No	3 (33%)
Cancer diagnosis	
Breast	5 (56%)
Thyroid	2 (22%)
Ovarian	2 (22%)
Time since diagnosis (yrs.) (mean = 1.89 yrs. )	
≤1	3 (33%)
2	3 (33%)
3	3 (33%)

## References

[1] Miller KD, Goding Sauer A, Ortiz AP, Fedewa SA, Pinheiro PS, Tortolero-Luna G, (2018) Cancer Statistics for Hispanics/Latinos, 2018. CA: A Cancer Journal for Clinicians, 68, 425–445. 10.3322/caac.21494 30285281

[2] Zheng Z, Jemal A, Han X, Guy GP Jr., Li C, Davidoff AJ, (2019) Medical Financial Hardship among Cancer Survivors in the United States. Cancer, 125, 1737–1747. 10.1002/cncr.31913 30663039

[3] Herrera DG, Schiefelbein EL, Smith R, Rojas R, Mirchandani GG and McDonald JA (2012) Cervical Cancer Screening in the US-Mexico Border Region: A Binational Analysis. Maternal and Child Health Journal, 16, 298–306. 10.1007/s10995-012-1130-8 22965734 PMC4535702

[4] Yanez B, McGinty HL, Buitrago D, Ramirez AG and Penedo FJ (2016) Cancer Outcomes in Hispanics/Latinos in the United States: An Integrative Review and Conceptual Model of Determinants of Health. Journal of Latina/o Psychology, 4, 114–129. 10.1037/lat0000055 27429867 PMC4943845

[5] State Cancer Profiles (2019) Incidence Rate Report by State for All Cancer Sites (All Stages) (2012–2016) in Hispanic (Any Race) Females Ages<50.

[6] Aguado Loi CX, Baldwin JA, McDermott RJ, McMillan S, Martinez Tyson D, Yampolskaya S, (2013) Risk Factors Associated with Increased Depressive Symptoms among Latinas Diagnosed with Breast Cancer within 5 Years of Survivorship. Psycho-Oncology, 22, 2779–2788. 10.1002/pon.3357 24000126

[7] Nápoles AM, Ortíz C, O’Brien H, Sereno AB and Kaplan CP (2011) Coping Resources and Self-Rated Health among Latina Breast Cancer Survivors. Oncology Nursing Forum, 38, 523–531. 10.1188/11.ONF.523-531 21875840 PMC3556482

[8] Umezawa Y, Lu Q, You J, Kagawa-Singer M, Leake B and Maly RC (2012) Belief in Divine Control, Coping, and Race/Ethnicity among Older Women with Breast Cancer. Annals of Behavioral Medicine, 44, 21–32. 10.1007/s12160-012-9358-5 22529040 PMC3873338

[9] Culver JL, Arena PL, Wimberly SR, Antoni MH and Carver CS (2004) Coping among African-American, Hispanic, and Non-Hispanic White Women Recently Treated for Early Stage Breast Cancer. Psychology & Health, 19, 157–166. 10.1080/08870440310001652669

[10] Janz NK, Friese CR, Li Y, Graff JJ, Hamilton AS and Hawley ST (2014) Emotional Well-Being Years Post-Treatment for Breast Cancer: Prospective, Multi-Ethnic, and Population-Based Analysis. Journal of Cancer Survivorship, 8, 131–142. 10.1007/s11764-013-0309-3 24222081 PMC4040232

[11] Buki LP, Garcés DM, Hinestrosa MC, Kogan L, Carrillo IY and French B (2008) Latina Breast Cancer Survivors’ Lived Experiences: Diagnosis, Treatment, and Beyond. Cultural Diversity and Ethnic Minority Psychology, 14, 163–167. 10.1037/1099-9809.14.2.163 18426289

[12] Ashing-Giwa KT, Padilla GV, Bohorquez DE, Tejero JS, Garcia M and Meyers EA (2006) Survivorship: A Qualitative Investigation of Latinas Diagnosed with Cervical Cancer. Journal of Psychosocial Oncology, 24, 53–88. 10.1300/J077v24n04_04 17182477

[13] Collins D, Villagran MM and Sparks L (2008) Crossing Borders, Crossing Cultures: Barriers to Communication about Cancer Prevention and Treatment along the U.S./Mexico Border. Patient Education and Counseling, 71, 333–339. 10.1016/j.pec.2008.03.013 18436416

[14] Owens B, Jackson M and Berndt A (2009) Complementary Therapy Used by Hispanic Women During Treatment for Breast Cancer. Journal of Holistic Nursing, 27, 167–176. 10.1177/0898010108330801 19372389

[15] Graves KD, Jensen RE, Cañar J, Perret-Gentil M, Leventhal K-G, Gonzalez F, (2012) Through the Lens of Culture: Quality of Life among La-tina Breast Cancer Survivors. Breast Cancer Research and Treatment, 136, 603–613. 10.1007/s10549-012-2291-2 23085764 PMC3547364

[16] Carrion IV, Nedjat-Haiem F and Macip-Billbe M (2017) “I Told Myself to Stay Positive” Perceptions of Coping among Latinos With a Cancer Diagnosis Living in the United States. American Journal of Hospice and Palliative Medicine, 34, 233–240. 10.1177/1049909115625955 26764346

[17] Lopez-Class M, Perret-Gentil M, Kreling B, Caicedo L, Mandelblatt J and Graves KD (2011) Quality of Life among Immigrant Latina Breast Cancer Survivors: Realities of Culture and Enhancing Cancer Care. Journal of Cancer Education 26, 724–733. 10.1007/s13187-011-0249-4 21706194 PMC3286609

[18] Martinez Tyson D, Jacobsen P and Meade CD (2016) Understanding the Stress Management Needs and Preferences of Latinas Undergoing Chemotherapy. Journal of Cancer Education, 31, 633–639. 10.1007/s13187-015-0844-x 25952939

[19] Ares I, Lebel S and Bielajew C (2014) The Impact of Motherhood on Perceived Stress, Illness Intrusiveness and Fear of Cancer Recurrence in Young Breast Cancer Survivors over Time. Psychology & Health, 29, 651–670. 10.1080/08870446.2014.881998 24410202

[20] Thomas DR (2006) A General Inductive Approach for Analyzing Qualitative Evaluation Data. American Journal of Evaluation, 27, 237–246. 10.1177/1098214005283748

[21] Lewis FM and Deal LW (1995) Balancing Our Lives: A Study of the Married Couple’s Experience with Breast Cancer Recurrence. Oncology Nursing Forum, 22, 943–953. 7567612

[22] Glaser BG and Strauss AL (1987) The Discovery of Grounded Theory: Strategies for Qualitative Research. Nursing Research, 17, 364. 10.1097/00006199-196807000-00014

[23] Lincoln YS and Guba EG (1985) Naturalistic Inquiry. SAGE, Newbury Park.

[24] Palomino H, Peacher D, Ko E, Woodruff SI and Watson M (2017) Barriers and Challenges of Cancer Patients and Their Experience with Patient Navigators in the Rural US/Mexico Border Region. Journal of Cancer Education, 32, 112–118. 10.1007/s13187-015-0906-0 26362872

[25] Semple CJ and McCance T (2010) Parents’ Experience of Cancer Who Have Young Children: A Literature Review. Cancer Nursing, 33, 110–118. 10.1097/NCC.0b013e3181c024bb 20142737

[26] Gonzalez P, Nuñez A, Wang-Letzkus M, Lim J-W, Flores KF and Nápoles AM (2016) Coping with Breast Cancer: Reflections from Chinese American, Korean American, and Mexican American Women. Health Psychology, 35, 19–28. 10.1037/hea0000263 26389720 PMC4695243

[27] Nápoles-Springer AM, Ortíz C, O’Brien H, Díaz-Méndez M and Pérez-Stable E (2007) Use of Cancer Support Groups among Latina Breast Cancer Survivors. Journal of Cancer Survivorship, 1, 193–204. 10.1007/s11764-007-0029-7 18648970

[28] Cuellar I, Arnold B and Gonzalez G (1995) Cognitive Referents of Acculturation: Assessment of Cultural Constructs in Mexican Americans. Journal of Community Psychology, 23, 339–356. 10.1002/1520-6629(199510)23:4&lt;339::AID-JCOP2290230406&gt;3.0.CO;2-7

